# A reversal of fortunes: climate change ‘winners’ and ‘losers’ in Antarctic Peninsula penguins

**DOI:** 10.1038/srep05024

**Published:** 2014-06-12

**Authors:** Gemma V. Clucas, Michael J. Dunn, Gareth Dyke, Steven D. Emslie, Hila Levy, Ron Naveen, Michael J. Polito, Oliver G. Pybus, Alex D. Rogers, Tom Hart

**Affiliations:** 1 Ocean and Earth Sciences, National Oceanography Centre, University of Southampton Waterfront Campus, European Way, Southampton, SO14 3ZH, UK; 2 Department of Zoology, South Parks Road, Oxford, OX1 3PS, UK; 3 British Antarctic Survey, High Cross, Madingley Road, Cambridge, CB3 0ET, UK; 4 Department of Biology and Marine Biology, University of North Carolina Wilmington, Wilmington, NC, 28403, USA; 5 USAF, Air Force Institute of Technology, 2950 Hobson Way,WPAFB, OH 45433-7765; 6 Oceanites Inc, PO Box 15259, Chevy Chase, MD 20825, USA; 7 Biology Department, Woods Hole Oceanographic Institution, Woods Hole, MA, 02543, USA

## Abstract

Climate change is a major threat to global biodiversity. Antarctic ecosystems are no exception. Investigating past species responses to climatic events can distinguish natural from anthropogenic impacts. Climate change produces ‘winners’, species that benefit from these events and ‘losers’, species that decline or become extinct. Using molecular techniques, we assess the demographic history and population structure of *Pygoscelis* penguins in the Scotia Arc related to climate warming after the Last Glacial Maximum (LGM). All three pygoscelid penguins responded positively to post-LGM warming by expanding from glacial refugia, with those breeding at higher latitudes expanding most. Northern (*Pygoscelis papua papua*) and Southern (*Pygoscelis papua ellsworthii*) gentoo sub-species likely diverged during the LGM. Comparing historical responses with the literature on current trends, we see Southern gentoo penguins are responding to current warming as they did during post-LGM warming, expanding their range southwards. Conversely, Adélie and chinstrap penguins are experiencing a ‘reversal of fortunes’ as they are now declining in the Antarctic Peninsula, the opposite of their response to post-LGM warming. This suggests current climate warming has decoupled historic population responses in the Antarctic Peninsula, favoring generalist gentoo penguins as climate change ‘winners’, while Adélie and chinstrap penguins have become climate change ‘losers’.

Climate warming around the western Antarctic Peninsula and in west Antarctica is amongst the fastest observed anywhere on Earth^1^,^2^,^3^. Changes in species' phenology, ranges and abundances have occurred over the past few decades^4^,^5^,^6^,^7^ but predicting these responses is complex as they occur at all trophic levels alongside changes in the abiotic environment. For example, the extent and duration of sea ice in the region is declining^8^ with correlated reductions in Antarctic krill^6^ (*Euphausia superba*), the main prey item for most meso- and top-predators in the Antarctic ecosystem. However the Antarctic climate has oscillated dramatically throughout the last 50 million years (Myr). The rate of current warming is highly unusual but not unprecedented for the Holocene period^9^ and the Pleistocene has been characterized by large-scale oscillations in global climate such as the 100,000 year cycles of ice ages^10^. During glacial periods, the Antarctic and sub-Antarctic ice sheets expanded and permanent sea ice was much more extensive^11^.

Understanding how past climate change has affected populations is critical for distinguishing between natural and anthropogenic impacts, especially in polar regions^12^. It can also help predict probable responses to future climate change - “looking backwards to look forwards”^13^. Molecular techniques allow us to identify major events in the evolutionary and demographic history of species and populations, thus revealing how climatic events have shaped the distribution and abundance of species through time. As such, it is possible to identify species as either climate change ‘winners’, with populations that remain stable or expand during these events, or climate change ‘losers’: species that decline in abundance and distribution or become extinct. Molecular techniques can also map the distribution of biodiversity at the sub-specific level. The maintenance of genetic diversity underpins conservation genetics^14^, and is a key priority of the Convention on Biological Diversity, albeit seldom measured^15^.

Here we use molecular techniques to characterize the demographic history and population structure of the *Pygoscelis* penguins breeding around the Antarctic Peninsula and Scotia Arc. We specifically investigate how climate change associated with the end of the last glacial period affected *Pygoscelis* penguin populations, and we then compare these results with analyses from the literature about their responses to current climate change. Adélie (*Pygoscelis adéliae*), chinstrap (*P. antarctica*) and gentoo (*P. papua*) penguins are sympatric in this region, with overlapping breeding colonies in some areas and all three species show high levels of breeding site fidelity^16^. In this region, Adélie and chinstrap penguins have a diet dominated by Antarctic krill during the breeding season, whilst gentoo penguins have a more variable diet feeding on varying proportions of krill, fish and small amounts of squid^17^. *Pygoscelis* penguins are important meso-predators in the marine food web and thus are sensitive indicators of environmental change, already showing responses to current climate warming^5^,^18^. Indeed, in one colony in the Ross Sea, Adélie penguins appear to be climate change ‘winners’ as warming is creating more nesting habitat as glacial ice fields retreat^19^.

## Results

### Population structure

**Figure 1:**
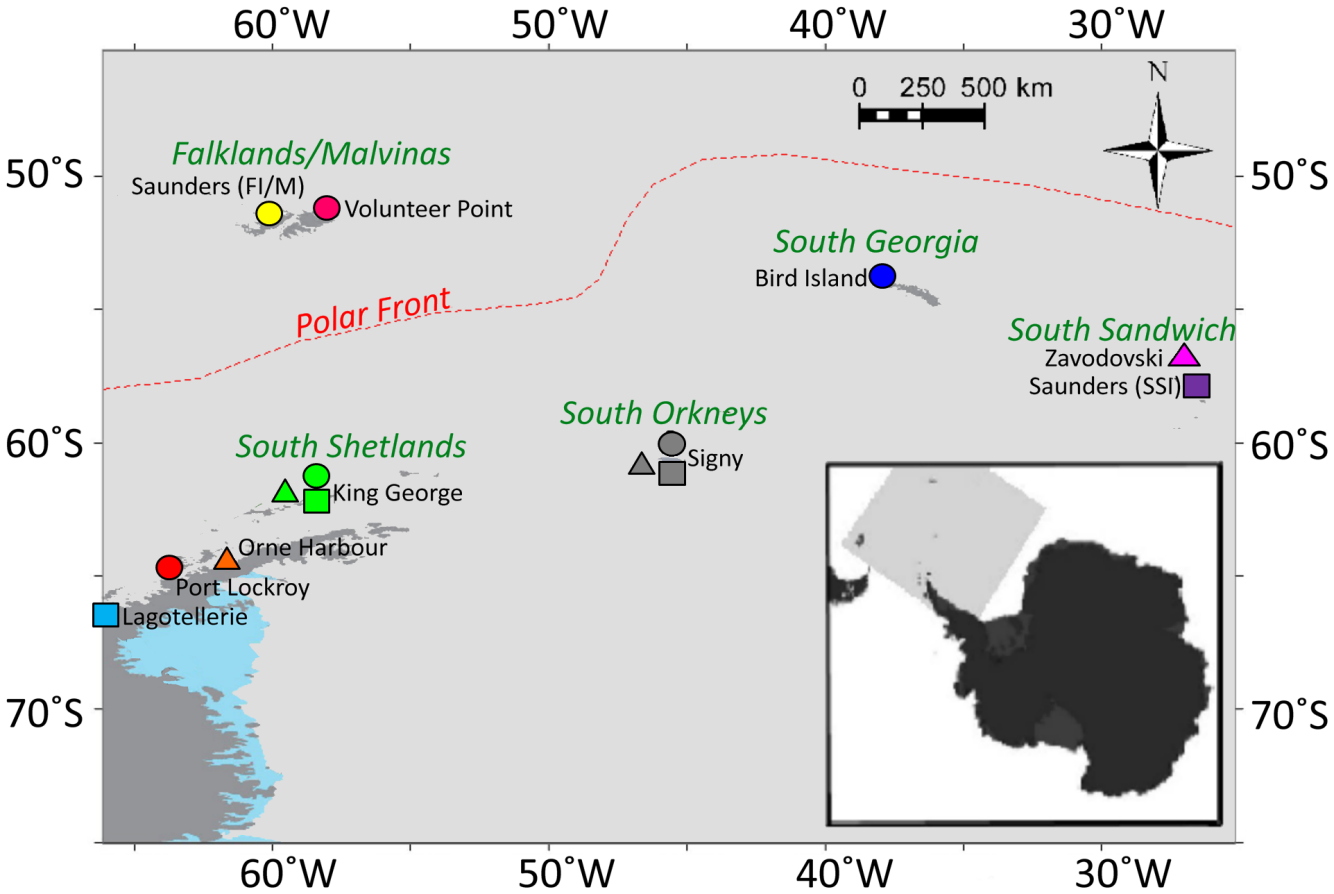
Sample locations across the Scotia Arc. Insert shows the location of the map relative to the Antarctic continent and South America. Gentoo penguin sample locations are shown with circles, chinstrap penguin colonies with triangles and Adélie penguin colonies with squares. Each sample location is coloured independently, and is consistent with Figure 2. The archipelago names are given in green. The map was produced by TH with help from Dr. Heather Lynch using ArcGIS and modified in ArcSoft® PhotoStudio.

We sequenced a fragment of the hypervariable region of the mitochondrial control region (HVR1) from colonies of each species spanning their entire latitudinal ranges and main breeding sites around the Antarctic Peninsula and Scotia Arc (Fig. 1). We sequenced a 316 base pair (bp) fragment from 249 gentoo penguins, a 465 bp fragment from 166 chinstrap penguins and a 601 bp fragment from 122 Adélie penguins (Table 1 and Supplementary Table S1 online; GenBank accession numbers: KJ646026-KJ646562). Although the length of the fragment sequenced in gentoo penguins was short compared to most studies of avian phylogeography, the proportion of variable sites was very high, giving sufficient information content for this study.

We detected significant population structure in gentoo penguins (*Φ_ST_* = 0.62, *p* = 0.000) with all colonies being genetically differentiated from one another (Supplementary Table S2 online) and showing isolation by distance (*r* = 0.63, *p* = 0.003). There was weak but significant population structure in chinstrap penguins (*Φ_ST_* = 0.027, *p* = 0.002) with just one colony, Zavadovski, showing genetic differentiation from the others (Supplementary Table S3 online) with no isolation by distance (*r* = 0.84, *p* = 0.084). We detected no population structure in Adélie penguins (*Φ_ST_* = 0.007, *p* = 0.07, Supplementary Table S4 online) despite sequencing the longest HVR1 fragment in this species, thus making our results robust to variations in fragment length.

**Figure 2:**
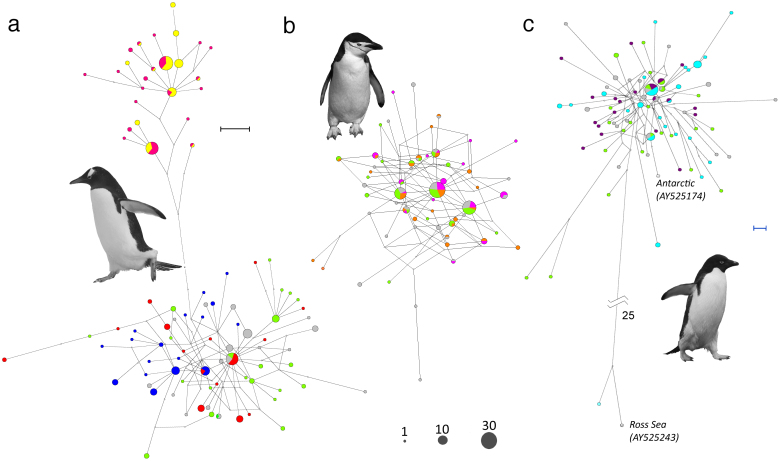
Median-joining haplotype networks for gentoo (a), chinstrap (b) and Adélie penguins (c). The area of each pie chart represents the number of haplotypes as shown by the scale at the bottom. Star contraction has been applied to the chinstrap penguin haplotype network and so some of the terminal nodes are not displayed. The representatives of the “Ross Sea” and “Antarctic” lineages (with GenBank accession numbers) are indicated on the Adélie network. Colours represent the populations where the haplotype was sampled, according to symbols on Figure 1. Black scale bar shows one mutation in gentoo and chinstrap penguins; blue scale bar shows one mutation in Adélie penguins; broken line shows 25 mutational steps. Photographic images belong to TH.

There was significant hierarchical population structure within gentoo penguins: when colonies were grouped into Falkland Island colonies versus all other colonies, 68.9% of the genetic variation was explained by the difference between these groups (AMOVA, among groups variation = 68.9%). The haplotype network for gentoo penguins (Fig. 2A) shows that there are two distinct monophyletic lineages that do not overlap geographically. One monophyletic lineage is found in the Falkland Islands and the other corresponds to haplotypes found in colonies south of the Polar Front. These two gentoo penguin lineages have previously been classified into two sub-species based on morphological differences: Northern gentoos (*Pygoscelis papua papua*) in the Falkland Islands and Southern gentoos (*Pygoscelis papua ellsworthii*) further south^20^,^21^.

Using a Bayesian coalescent framework implemented in BEAST we estimated the time to the most recent common ancestor of Northern and Southern gentoo penguins. To calibrate the genealogy we used the rate of molecular evolution of the HVR1 region estimated for Adélie^22^ and Northern rockhopper penguins (*Eudyptes moseleyi*)^23^. These calibrations date the divergence to have occurred 25 kyr ago (95% HPD = 11–42 kya) and 44 kyr ago (95% HPD = 30–59 kya), respectively.

### Demographic histories with respect to climate

**Figure 3:**
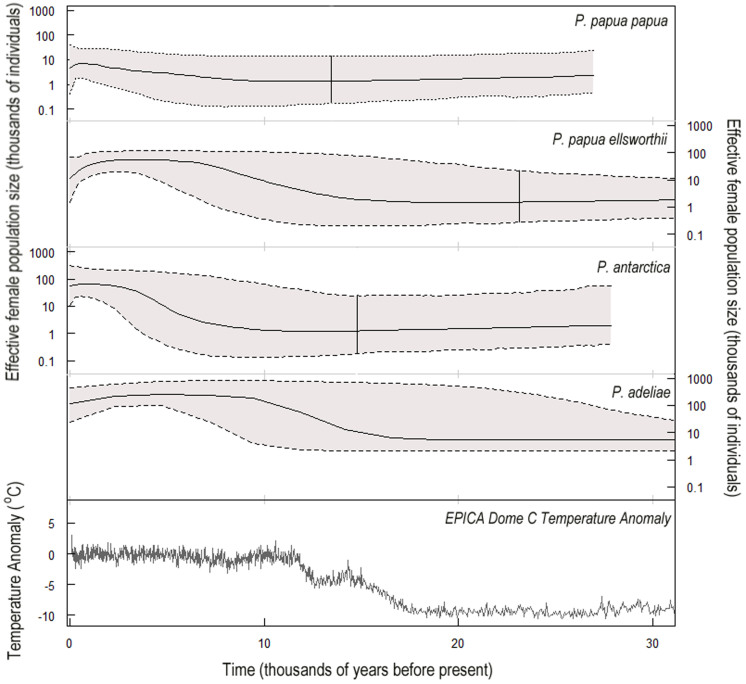
Bayesian skyline plots showing the change in effective female population size for each species and sub-species. Solid lines show the median estimate; dotted lines show the 95% highest posterior density interval. Solid vertical line shows the mean tMRCA for the population, whilst the projection is made to the upper limit of the 95% highest posterior density interval of the tMRCA. The bottom panel shows the Antarctic temperature anomaly (the difference from the average of the last 1000 years) as estimated from the EPICA Dome C ice core^54^.

All three species have undergone demographic expansions during the Holocene, as demonstrated by their star-shaped haplotype networks (Fig. 2), uni-modal mismatch distributions (Supplementary Fig. S1 online) and significantly negative values of Fu's *F_S_* statistic (Table 1). However the extent of their demographic expansions appears to have been mediated by their latitudinal distributions. This mediation is reflected in the values of Tajima's *D* test statistics, which become more negative the further south the species is distributed (Table 1). Bayesian skyline plots, which show the effective female population sizes over time, also show this latitudinal pattern (Fig. 3). Northern gentoos, breeding the furthest north, have expanded the least, Southern gentoos which breed slightly further south have expanded to a greater extent and chinstraps and Adélies, breeding the furthest south, have expanded the most. Figure 3 (bottom panel) also plots Antarctic temperature anomalies for the past 30 kyr. The population expansions all occur following the climatic warming that occurred after the last LGM, suggesting that the populations were expanding out of glacial refugia.

## Discussion

The differences in the degrees of population structure in these species may be explained by their different dispersal behaviors in the austral winter. Gentoo penguins are resident at or near their colonies in winter whilst chinstrap and Adélie penguins are more dispersive, often travelling hundreds to thousands of kilometers in the winter to forage at the pack ice edge^16^,^17^. Winter dispersal has been shown to be an important determinant of population structure in seabirds^24^ and the patterns observed here are in agreement with the majority of seabirds studied thus far; those that are residents in winter show higher levels of population structure than more migratory species. It is important to note here that we are investigating population structure at the regional, not the local scale. At the local scale, where colonies are separated by tens of kilometers or are within the same archipelago, we would not expect to find population structure as members of all three of these species have been observed visiting nearby colonies at low rates^19^,^25^,^26^,^27^ and chinstrap penguins lack population structure at these scales^28^. Changes in the local conditions, such as increased sea ice or ice-bergs which block access to colonies, has been found to increase the chances of individual Adélie penguins visiting nearby colonies^25^. However it seems like the long migrations undertaken by chinstrap and Adélie penguins during the winter must facilitate gene flow at the regional level, whilst the lack of long migrations in gentoo penguins creates the population structure we have observed.

The Polar Front acts as a barrier to gene flow in many diverse marine taxa^29^ because of the abrupt change in ecological conditions that it represents: from the relatively warm waters of the southern Atlantic, Pacific or Indian Ocean to the cold waters of the Antarctic Circumpolar Current. The Polar Front may also act as a barrier to gene flow in gentoo penguins as shown by the monophyletic lineages observed either side of it. Using rates of molecular evolution as calibrations, we estimate that the two sub-species diverged from one another between 11 and 59 kya. This means the divergence most likely occurred during the last glacial period or just after it. Lineage divergence may have occurred for two reasons. Firstly, the populations could have been isolated from one another within different glacial refugia. Long-term isolation of populations from one another results in genetic differentiation through genetic drift, and this generates distinct genetic lineages^30^. Secondly, they may have diverged from one another following glacial retreat as more habitat became available. Southern gentoos may have migrated out of a single gentoo refuge to colonize areas south of the Polar Front as the ice retreated. Lineage divergence during the last glacial period is also evident in Adélie penguins in the Ross Sea. Ritchie and colleagues^31^ identified two mitochondrial DNA lineages of Adélie penguins which diverged from one another approximately 75 kya, and they suggest that limited breeding opportunities during the last glaciation separated the two lineages from one another in glacial refugia. Thus climate change in Antarctica appears to have been a strong driver of micro-evolutionary change.

Past climate change has also had a serious effect on the population sizes of the *Pygoscelis* penguins. We have shown strong evidence that these species were expanding southwards out of glacial refugia as the ice retreated after the last glacial maximum (LGM, ca 19.5–16 kya^32^), as those species which breed at higher latitudes were able to recolonize the most habitat as it became available. All three species require ice-free ground to build their nests on as well as open water in the vicinity, allowing them to travel to and from their foraging grounds during the breeding season. During the LGM, Antarctica was encircled by 100% more winter sea ice than today^32^ and although summer sea ice extents are largely unknown, permanent and thick sea ice most likely surrounded the entire continent. A few polynyas persisted, but these probably would not have supported penguins (ref^33^, Sven Thatje pers. comm. 2012). Thus all the *Pygoscelis* penguins would have been forced to move further north onto islands and other land-masses that remained unglaciated and free from permanent sea ice, or were exposed by the lower sea level. It is interesting to note that sea levels were 120 m lower at the LGM compared to today^11^, and so ice-age colony sites are now probably submerged.

As the climate warmed after the LGM (Figure 3, bottom panel), the extent and duration of winter sea ice declined and ice shelves retreated, allowing *Pygoscelis* penguins to expand as more habitats became available to the south. This impact is similar to current climate change, which is also reducing the extent and duration of winter sea ice around the western Antarctic Peninsula^8^. This current warming is benefitting Southern gentoo penguins, as they are expanding their range southwards and increasing in number, especially at their more southerly colonies^5^. This mirrors the pattern we detected in response to warming after the LGM: Southern gentoos expanded more than Northern gentoos. However chinstrap and Adélie penguins in the Antarctic Peninsula are currently in decline. The reasons for these declines are debated but the abundance of these two species appears to be closely linked to the availability^18^ and recruitment^27^ of Antarctic krill, their main prey. Adélie and chinstrap penguins showed population increases during the first part of the 20^th^ century when climatic conditions were favorable for krill and the harvesting of marine mammals reduced competition between penguins and other krill predators^34^,^35^,^36^. This has been followed by chinstrap and Adélie population declines, when sea ice reductions resulted in krill declines^18^. Declines in Adélie penguins may have been exacerbated by declines in Antarctic silverfish, which are also a component of Adélie penguin diets and require sea ice for protection during larval phases^7^,^37^. However, others argue that krill stocks are sufficient for Adélie penguins, and there are suggestions that other factors such as snow accumulation and increased melt-water run-off are responsible for declining breeding success^38^,^39^. There is also evidence that in the southern sector of the Antarctic Peninsula some Adélie penguin colonies are increasing, whilst others are decreasing. Differences in population dynamics over relatively small spatial scales in this region mean that identifying a trend is difficult. However, overall it seems that climate warming is no longer benefitting all three *Pygoscelis* penguins in the Antarctic Peninsula by creating more suitable breeding habitat as it did after the LGM, but it is only benefitting the more opportunistic and generalist gentoo penguin, whose diverse and flexible foraging niche^40^,^41^ likely make this species relatively less sensitive to declines in krill.

This ‘reversal of fortunes’ for two former climate change ‘winners’ has resulted from anthropogenic impacts outside the range of natural variation that has occurred in the past. Rapid warming trends in the Antarctic Peninsula over the past 50 years has led to decreased sea ice, loss of winter habitat, and a reduction in krill stocks that is negatively affecting Adélie and chinstrap penguins, but not gentoo penguins^5^,^18^, which apparently are not as reliant on krill^17^. While we know of no other examples of ‘reversal in fortunes’ as documented here, we expect many more will be identified as global warming proceeds and biodiversity declines.

## Methods

### Sample collection

Shed penguin feathers were collected from Volunteer Point and Saunders Island in May 2010 and from Port Lockroy, Orne Harbour and Lagotellerie over three field seasons from 2009 to 2012. When collecting shed feathers, 80–125 molted penguin body and tail feathers were collected with feathers being collected at least 2 meters apart to minimize the chance of obtaining duplicate samples from an individual. Feathers were stored dry at ambient temperature until extraction.

Where direct samples were taken (Bird, Zavodovski, Saunders (SSI), King George and Signy Islands), birds were seized with both hands by the upper body and the flippers were restrained by the same handler. The head was placed under the arm of the handler to stop the bird biting in accordance with the literature on minimizing stress in restrained penguins^42^,^43^. The second person plucked two feathers from this bird's lower back or took blood samples. Where taken, bloods were from the brachial vein using a 25 G needle and syringe, and were immediately stored in 95% ethanol at ambient temperature. The animal was then released at the edge of the colony. It is possible to pluck feathers with a minimum of stress within 30 seconds, but usually no longer than 2 minutes. Blood samples usually take 2–3 minutes of restraint. Only 40 gentoo penguin adult blood samples were used in this study, previously obtained from Bird Island, South Georgia by researchers from the British Antarctic Survey. All other direct samples were plucked feathers.

Direct sampling was conducted under permits from the Falkland Islands Environmental Planning Department, The Government of South Georgia and the South Sandwich Islands, the UK Foreign and Commonwealth Office, and the US National Science Foundation. Each of these permits was issued following independent ethical review of the sampling. There are no legal restrictions covering research on animals in South Georgia or Antarctica. However, all sampling was carried out in accordance with UK Home Office guidelines and received ethical approval from the University of Oxford, the Zoological Society of London and the University of North Carolina, Wilmington. Blood sampling at Bird Island received ethical approval from the British Antarctic Survey.

### DNA extraction and amplification

Feathers were prepared by finely slicing the proximal 3 mm of the feather calamus and any attached tissue for DNA extraction. Where tail feathers were available, the calamus was further sliced open and 2 mm of the blood capillary was sampled in addition to the proximal end of the calamus. Genomic DNA (gDNA) was extracted from feather fragments and blood using DNeasy Blood and Tissue Kits (http://www.qiagen.com/) according to the manufacturer's instructions for animal tissue, with the following modification to the incubation step for feather samples: 40 μl of proteinase K and 180 μl buffer ATL was added to the tissue and incubation was extended to 48 hours at 56°C.

The hypervariable region 1 (HVR-1) of the mtDNA genome was amplified from chinstrap penguin gDNA using the primers L-tRNA^glu^ and H-A650 (ref. 31,44). The primer AP1STR (5′-CCACCCTATACATACAATTCCCCTCCC-3′) was designed using Primer3 (http://primer3.wi.mit.edu/) from sequences published on GenBank to amplify the Adélie HVR-1 region paired with H-A650. The primers GPPAIR3F (5′-TTCACGTGAGGAGCCCGACCA-3′) and GPPAIR3R (5′-CTCAGGGCTAAACGGGAACTCTGC-3′) were designed in the same way to amplify the gentoo HVR-1 region. The PCR reaction mix consisted of 7.5 μl Qiagen Taq PCR Master Mix, 2 nM primers, approximately 10 ng of Adélie or gentoo gDNA, or 1 ng chinstrap gDNA, made up to a final volume of 15 μl with Milli-Q water. The thermocycling conditions for Adélie and chinstrap reactions were: 94°C for 3 minutes; 40 cycles of 94°C for 45 seconds, 52.5°C for 45 seconds and 72°C for 1 minute; followed by a 10 minute extension period at 72°C. The thermocycling conditions for the gentoo penguin amplifications were the same but the annealing temperature was raised to 54°C.

PCR products were purified and sequenced in both directions using the EZ-Seq service offered by Macrogen Europe (http://www.macrogen.com/). The same primers from the PCR amplification were used for sequencing, with the exception that chinstrap PCR products were sequenced with H-A650 and CPSEQLHS2 (5′-TTAGGGTTGTTATTGTACTCTGGA-3′). CPSEQLHS2 was designed using Primer3 from the sequences generated with H-A650, because L-tRNA^glu^ was found to be problematic when used in the chinstrap sequencing reaction.

Geneious Basic v5.6.4, created by Biomatters (http://www.geneious.com), was used to align forward and reverse sequences and extract a consensus sequence. When two fluorescent signals were observed at a single base position, as a result of heteroplasmy^22^, these sites were treated as missing data.

### Data analysis

Arlequin v3.5 (ref. 45) was used to calculate standard molecular diversity indices and pairwise *Φ_ST_*s, to perform Mantel tests for isolation by distance, analyses of molecular variance (AMOVAs), neutrality tests and to calculate mismatch distributions. Molecular diversity measures and molecular distances were calculated where possible with the Tamura correction for unequal base frequencies and a gamma distribution model of substitution rate heterogeneity among sites. The shape parameter (*α*) of the gamma distribution was 0.102, 0.01 and 0.125 for gentoos, chinstraps and Adélies, respectively, as calculated in jModelTest v0.1.1 (ref. 46,47). Pairwise *Φ_ST_*s were calculated between all colonies within species and significance was determined using 10,000 permutations of haplotypes between colonies, followed by the Bonferroni correction for multiple comparisons. Mantel tests for isolation by distance were performed by calculating the shortest at sea route between colonies using Google Earth v6.1 (http://earth.google.co.uk). Significance was determined through 10,000 permutations of the data. AMOVAs were used to look for hierarchical population structure. Population structures tested in gentoo penguins were: (A) no grouping of populations; (B) populations divided into Falkland Island populations (*P. p. papua*) and non-Falkland Island populations (*P. p. ellsworthii*); and (C) populations divided into *P. p. papua*, Bird Island gentoos and all other *P. p. ellsworthii*. Given the high degree of divergence found between *P. p. papua* and *P. p. ellsworthii*, AMOVAs were repeated for the *P. p. ellsworthii* populations with the following structures: (A) no population groupings; and (B) populations divided into Bird Island gentoos versus all other gentoos. Population structures tested in chinstrap penguins were: (A) no grouping of populations; and (B) populations divided into South Sandwich Island chinstraps (Zavodovzki) versus all other chinstraps. Structures tested in Adélie penguins were: (A) no grouping of populations; (B) populations grouped into South Sandwich Islands (Saunders SSI) versus Antarctic populations; and (C) populations grouped into South Sandwich Islands (Saunders SSI), northern Antarctic populations (King George Island and Signy Island) and southern Antarctic populations (Lagotellerie). 95% confidence intervals were calculated using 5,000 bootstrap replicates. Tajima's *D* and Fu's *F*_S_ statistics were calculated for the entire species or sub-species (Table 1) and for each individual colony (Supplementary Table S1).

Median joining haplotype networks were drawn in Network v4.6.1.0 (http://www.fluxus-engineering.com). Because of the complexity of the chinstrap haplotype network, the star contraction option was used with a maximum star radius of five, to remove some of the terminal branches from the network. Members of the two Adélie penguin lineages identified previously were taken from GenBank (accession numbers AY525423 and AY525174) and included in the Adélie haplotype network for comparison.

To date the divergence of *P. p. papua* and *P. p. ellsworthii*, the time to their most recent common ancestor (T_MRCA_) was estimated using the Bayesian MCMC approach implemented in BEAST v1.7.2 (ref. 48). A demographic model of exponential population growth was used and jModelTest combined with Bayes factor (BF) analysis was used to select the K2P + Γ substitution model (after all models with invariant sites were excluded). When the Bayesian Inference Criterion implemented in jModelTest supported more than one substitution model, model support was evaluated using Bayes factors^49^. The marginal likelihoods for the Bayes factor calculations were estimated under each model using both the path sampling (PS) and stepping stone sampling (SS) methods implemented in BEAST using 100 million generations and a burn-in of 10%. Statistical support was then evaluated using log (BF) using both PS and SS methods as per Kass & Raftery^50^. In the case of log (BF) < 1, which does not constitute support for either model, the simpler substitution model was selected for the main analysis. The genealogy was calibrated using two different rates of molecular evolution under a strict molecular clock model: the rate of 0.86 substitutions/site/Myr was implemented as a normally distributed prior with a standard deviation of 0.2 to represent the uncertainty in this estimate^22^, whilst the rate of 0.35 s/s/Myr was implemented as a fixed rate as the uncertainty in this estimate was not published^23^. The MCMC chain was run for 100 million generations, sampling every 10,000 generations, with a burn-in of 10%. MCMC output was visualised in Tracer v1.5 (http://tree.bio.ed.ac.uk/software/tracer/) to check for convergence and mixing, and all effective sample sizes (ESSs) were >300. Each run was repeated at least four times to check for stationarity. To estimate the T_MRCA_, the results from at least three runs were combined in Tracer.

Bayesian skyline plots (BSPs)^51^, created using BEAST and Tracer, were used to investigate the historical population size of *P. p. papua*, *P. p. ellsworthii*, chinstrap and Adélie penguins. In all analyses, rate constancy between branches could not be rejected (the coefficient of variation under the uncorrelated relaxed molecular clock was not different from zero) and so strict clocks were used. All trees were calibrated with the rate of molecular evolution from Adélie penguin pedigrees as a normal prior (mean = 0.55 s/s/Myr, SD = 0.15)^22^. Weak (uniform) priors were specified for population size, with an upper limit of 1 × 10^10^ for the each population size parameter. jModelTest with the Bayesian Inference Criterion and Bayes factors were used to select the most appropriate substitution model for each dataset, as above. The models selected were the TN93 + G (ref. 52) model for Adélies and *P. p. ellsworthii* and the K80 + G (ref. 53) model for chinstraps and *P. p. papua*. The MCMC chain length and evaluation was described as above and the results from two or three runs were combined to make sure all ESSs were >300. A generation time of 8 years was assumed for all species. Where the shape and 95% highest posterior density (HPD) of the BSPs suggested that a constant population size through time was plausible, demographic models of exponential growth versus constant population size were compared using Bayes factors, keeping all other settings in the MCMC run the same. In all cases, the exponential growth model received greater support than a constant size model.

## Author Contributions

G.V.C. performed all lab and analytical work, and wrote the paper. T.H., S.D.E., M.J.D., M.J.P., H.L. and R.N. conducted field work and provided samples. O.G.P. provided analytical support with Bayesian coalescent inferences. T.H. designed the study and prepared all figures. G.D. and A.D.R. were also involved in study design. All authors discussed the study results and implications and contributed to editing the manuscript.

## Supplementary Material

Supplementary Information A reversal of fortunes: climate change ‘winners’ and ‘losers’ in Antarctic Peninsula penguins - Supplementary Information

## Figures and Tables

**Table 1:** mtDNA diversity and neutrality test results for each species and sub-species

	n	N_H_	N_P_	H (SD)	π (SD)	Fu's *F_S_*	Tajima's *D*
Gentoo penguin	249	110	58	0.981 (0.003)	0.023 (0.012)	−24.51***	−0.726
*P. p. papua*	91	40	22	0.955 (0.009)	0.008 (0.005)	−26.79***	−1.222
*P. p. ellsworthii*	158	70	48	0.984 (0.003)	0.012 (0.007)	−25.79***	−1.647*
Chinstrap penguin	166	116	46	0.987 (0.004)	0.006 (0.004)	−26.36***	−1.895**
Adélie penguin	122	115	128	0.999 (0.001)	0.016 (0.008)	−24.49***	−1.980**

n, number of individuals sequenced; N_H_, number of haplotypes; N_P_, number of polymorphic sites; H, haplotype diversity; π, nucleotide diversity; SD, standard deviation.

*denotes significance at α = 0.05;

**denotes significance at α = 0.01;

***denotes significance at α = 0.001.
