# Supplementary material for: A reversal of fortunes: climate change ‘winners’ and ‘losers’ in Antarctic Peninsula penguins
Source: Sci Rep. 2014 Jun 12;4:5024. doi: 10.1038/srep05024 (PMC4034736; doi:10.1038/srep05024)
Supplement: Supplementary Information — A reversal of fortunes: climate change ‘winners’ and ‘losers’ in Antarctic Peninsula penguins - Supplementary Information [file srep05024-s1.pdf]

A reversal of fortunes: climate change 'winners' and 'losers' in Antarctic Peninsula penguins

Supplementary Information

Authors: Gemma V. Clucas, Michael J. Dunn, Gareth Dyke, Steven D. Emslie, Hila Levy, Ron Naveen, Michael J. Polito, Oliver G. Pybus, Alex D. Rogers, and Tom Hart.

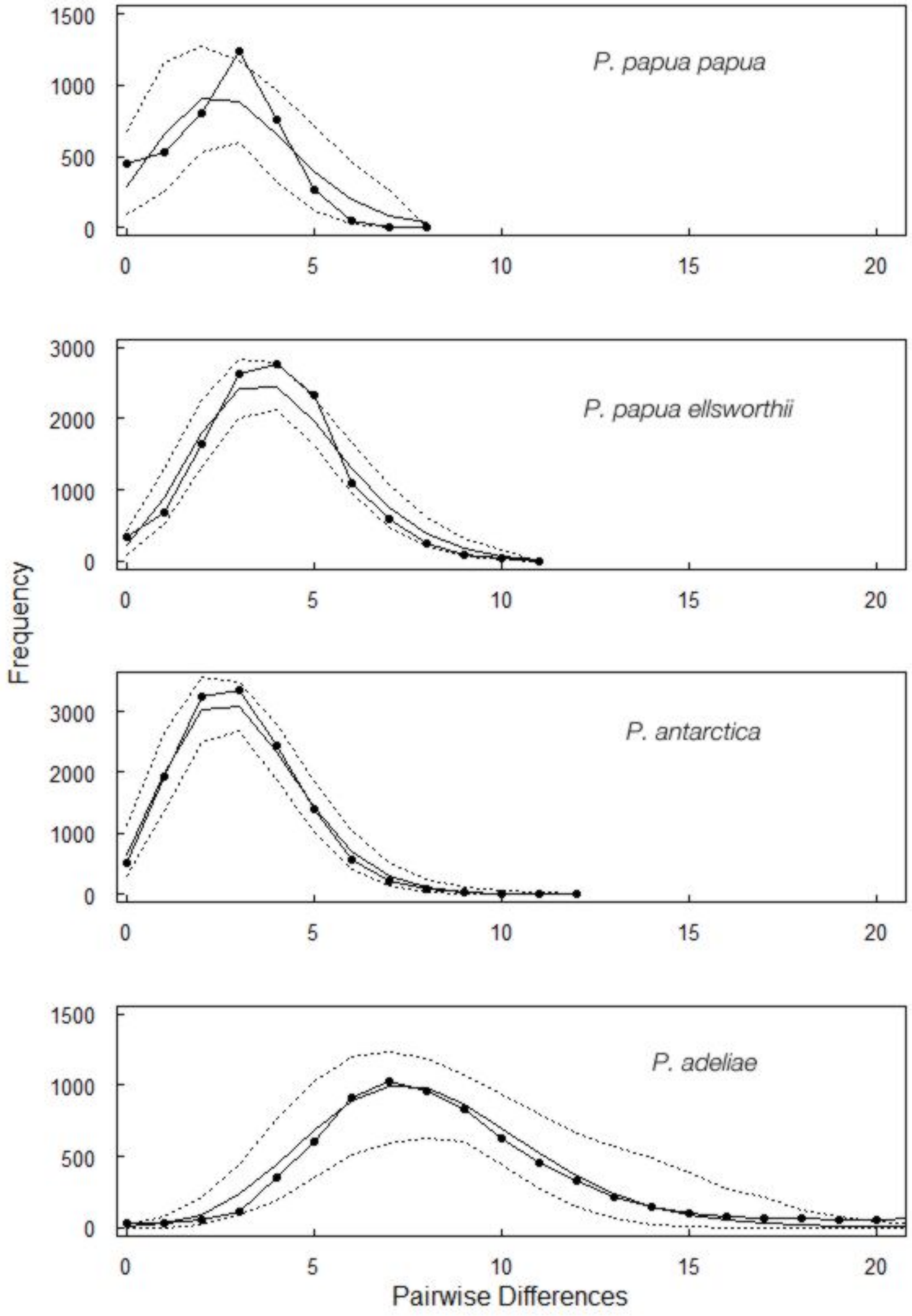

Figure S1. Mismatch distributions showing the unimodal shape indicative of population expansions. Solid line with circles, observed frequency; solid line, simulated frequency; dotted lines, 95% confidence interval.

Table S1. Measures of mtDNA diversity and neutrality test results for each species, for each sub-species and for each population. All analyses were performed with the Tamura correction for unequal base frequencies and a gamma distribution model of substitution rate heterogeneity among sites.

	n	N_H	N_P	H (SD)	π (SD)	Fu's F_s	Tajima's D
Gentoos	249	110	58	0.981 (0.003)	0.023 (0.012)	**-24.51*** **	-0.726
P. p. papua	91	40	22	0.955 (0.009)	0.008 (0.005)	**-26.79*** **	-1.222
P. p. ellsworthii	158	70	48	0.984 (0.003)	0.012 (0.007)	**-25.79*** **	**-1.647***
Saunders Island (FI/M)†	51	17	13	0.903 (0.021)	0.007 (0.005)	**-8.35*** **	-0.590
Volunteer Point †	40	23	19	0.923 (0.029)	0.008 (0.005)	**-19.02*** **	-1.343
Bird Island‡	38	19	18	0.935 (0.022)	0.008 (0.005)	**-11.68*** **	-1.164
Signy Island‡	42	19	23	0.934 (0.020)	0.011 (0.006)	**-8.96*** **	-1.281
King George Island‡	41	23	26	0.960 (0.015)	0.013 (0.008)	**-12.59*** **	-1.073
Port Lockroy‡	37	18	19	0.910 (0.030)	0.011 (0.006)	**-8.38*** **	-0.872
Chinstraps	166	116	46	0.987 (0.004)	0.006 (0.004)	**-26.36*** **	**-1.895** **
Zavodovski	36	30	18	0.989 (0.001)	0.006 (0.003)	**-17.04*** **	-1.366
Signy Island	44	35	25	0.987 (0.009)	0.007 (0.004)	**-20.78*** **	-1.450
King George Island	46	32	22	0.968 (0.014)	0.005 (0.003)	**-16.32*** **	**-1.631***
Orne Harbour	40	33	20	0.990 (0.008)	0.007 (0.004)	**-21.83*** **	-0.932
Adélies	122	115	128	0.999 (0.001)	0.016 (0.008)	**-24.49*** **	**-1.980** **
Saunders Island (SSI)	23	23	54	1.000 (0.013)	0.014 (0.007)	**-18.22*** **	**-1.764***
Signy Island	29	28	60	0.998 (0.010)	0.014 (0.007)	**-13.95*** **	**-1.680***
King George Island	28	27	68	0.997 (0.010)	0.017 (0.009)	**-15.20*** **	**-1.583***
Lagotellerie	42	37	84	0.991 (0.009)	0.016 (0.008)	**-13.69*** **	**-1.828***

n, number of individuals sequenced; N_H, number of haplotypes; N_P, number of polymorphic sites; H, haplotype diversity; π, nucleotide diversity; SD, standard deviation, * denotes significance at $\alpha = 0.05$; ** denotes significance at $\alpha = 0.01$; ***denotes significance at $\alpha = 0.001$; † denotes gentoo penguin populations belonging to the *P. p. papua* sub-species; ‡ denotes gentoo populations belonging to the *P. p. ellsworthii* sub-species.

Table S2. Pairwise Φ_{ST} values below the diagonal with associated *p*-values above the diagonal, calculated between all gentoo penguin colonies.

	Saunders Island	Volunteer Point	Bird Island	Signy Island	King George Island	Port Lockroy
Saunders Island		**0.002**	**<0.001**	**<0.001**	**<0.001**	**<0.001**
Volunteer Point	**0.066**		**<0.001**	**<0.001**	**<0.001**	**<0.001**
Bird Island	**0.775**	**0.750**		**<0.001**	**<0.001**	**<0.001**
Signy Island	**0.759**	**0.729**	**0.237**		**<0.001**	**<0.001**
King George Island	**0.742**	**0.711**	**0.234**	**0.081**		**<0.001**
Port Lockroy	**0.775**	**0.746**	**0.221**	**0.098**	**0.071**	

Bold denotes significance after Bonferroni correction for multiple comparisons.

Table S3. Pairwise Φ_{ST} values below the diagonal with associated *p*-values above the diagonal, calculated between all chinstrap penguin colonies.

	Zavodovski	Signy Island	King George Island	Orne Harbour
Zavodovski		**<0.001**	**0.002**	**<0.001**
Signy Island	**0.053**		0.136	0.056
King George Island	**0.046**	0.009		0.449
Orne Harbour	**0.049**	0.015	-0.001	

Bold denotes significance after Bonferroni correction for multiple comparisons.

Table S4. Pairwise Φ_{ST} values below the diagonal with associated *p*-values above the diagonal, calculated between all Adélie penguin colonies. None of the pairwise comparisons were significant after Bonferroni correction.

	Saunders SSI	Signy Island	King George Island	Lagotellerie
Saunders SSI		0.667	0.480	0.172
Signy Island	-0.005		0.682	0.040
King George Island	0.000	-0.005		0.055
Lagotellerie	0.008	0.015	0.014	
